# The Constitution of Man, Considered in Relation to External Objects

**Published:** 1835-10-01

**Authors:** 


### THE CONSTITUTION OF MAN, CONSIDERED IN RELATION TO EXTERNAL OBJECTS. Second Edition, corrected and enlarged, 8vo, pp. 458. Edinburgh and London, 1835.

ETIOLOGICAL science will derive great advantages from the principles propounded by Mr. Combe, in this improved edition of his "Constitution of Man." In accordance with the objects of this Journal, however, we resign the purely philosophical portion of these principles, to be studied and applied by moralists, legislators, and theologians; and, perceiving many excellent hygienal precepts, as well as physiological maxims, disseminated over the work, we shall select some of these, and earnestly recommend them to the attention of our medico-chirurgical readers, as a source in which they will find many instructive observations, available for the conservation of health and the prevention of disease.

Mr. Combe inculcates the topics discussed in this volume with the fervour of philanthropical eloquence, and with the solemnity due to investigations instituted for the generous end of distinguishing and purifying the elements of everlasting truth. He opens his work with a section of introductory remarks, intended to exhibit a view of the constitution of human nature,* and its relations to external objects. In this section, he endeavours to substantiate and apply a doctrine, which he regards as the key to the true theory of the divine government of the world—namely, *the independent existence and operation of the natural laws of creation.* He divides the natural laws into three great classes—PHYSICAL, ORGANIC, and MORAL; and the peculiarity of his doctrine is, that these laws operate independently of each other; that each of them requires obedience to itself; that each, in its own specific way, rewards obedience and punishes disobedience, and that human beings are happy, in proportion to the extent in which they place themselves in accordance with all of these divine institutions.

As an illustration of Mr. C.'s philosophy, we make an extract from his 26th page. There he remarks—

"The organic laws operate independently; and, hence, one individual who has inherited a fine bodily constitution from his parents, and observes the rules of temperance and exercise, will enjoy robust health, although he may cheat, lie, blaspheme, and murder his fellow-creature; while another, if he have inherited a feeble constitution, and disregards the rules of temperance and exercise, will suffer pain and sickness, although he may be a paragon of every Christian virtue. These results frequently occur in the world; and, on every such occasion, the darkness and inscrutable perplexity of the ways of Providence are generally moralized upon, or a future life is called in as the scene in which these crooked paths are to be made straight. But, if my views be correct, the divine wisdom and goodness are abundantly conspicuous in these events, for we perceive that, by this distinct operation of the organic and moral laws, order is preserved in creation, and the means of discipline and improvement are afforded to all the human faculties."

Mr. C. next enters upon an explanation of the natural laws. According to him, every natural object has received a definite constitution, in virtue of which it acts in a particular way; there must, therefore, be as many natural laws as there are distinct modes of action, of substances, and beings viewed by themselves. But, he adds, substances and beings stand in certain relations to each

---

* Mr. Combe published the first edition of his "Constitution of Man" in the year preceding the late Earl of Bridgewater's decease, and five years before the first of the "Bridgewater Treatises" made its appearance.

other, and modify each other's action in an established and definite manner, according to that relationship; wherefore, he concludes, there must be also as many laws of nature as there are relations between different substances and beings. He reckons it impossible, in the present state of knowledge, to elucidate all these laws; countless years may elapse before they shall be discovered. Under this conviction, he limits his inquiry to the physical laws, the organic laws, and the laws which characterize intelligent beings. The physical laws embrace all the phenomena of mere matter; he illustrates their operations by instances of chemical action, and the agency of gravitation. Organized substances and beings have properties peculiar to themselves—they act, and are acted upon, in conformity with their constitution, and are subject to the organic laws; their distinguishing characteristic is, that the individuals derive their existence from other organized beings, are nourished by food, and go through a regular process of growth and decay; they are classed into the two great subdivisions of animals and vegetables—the former constitute the subject of Mr. C.'s present observations. Intelligent beings embrace all animals that have distinct consciousness, from the lowest of the inferior creatures up to man—they are divided into two classes, the intelligent and animal, and the intelligent and moral creatures; the dog, horse, and elephant belong to the first class, because they possess some degree of intelligence and certain animal propensities, but no moral feelings—man belongs to the second, because he possesses all the three. The mental faculties have received a precise constitution—have been placed in fixed and definite relations to external objects, and act regularly; Mr. C., therefore, speaks of their acting according to determinate modes or rules, and these he calls the moral and intellectual laws; and, in considering the natural laws generally, he directs attention to four important principles: that these laws are independent of each other—that obedience to each of them is attended with its own reward, and disobedience with its own punishment—that they are universal, unbending, and invariable in their operation—and that they are in harmony with the constitution of man. We must resist the temptation to particularize the facts and arguments by which these positions are substantiated. Whoever studies them with candour will ensure a high reward.

His method leading him next to investigate the constitution of man, and the natural laws to which he is subjected, and to endeavour to discover how far the external world is arranged with wisdom and benevolence, in regard to the human race, Mr. C. divides this part of his inquiry into five separate sections. In these, he considers man as a physical being*—as an organized being—as an animal, moral, and intellectual being. He then compares the faculties of man with each other, and evinces the supremacy of the moral sentiments and the intellect over the animal propensities; and, last of all, he institutes a comparison between the human faculties and the external objects of creation. The next part of his inquiry is on the sources of human happiness, and the conditions requisite for maintaining it. He states, p. 98—

"The *first* and most obvious circumstance which attracts attention is, that all enjoyment must necessarily arise from *activity* of the various systems of which the human constitution is composed. The bones, muscles, nerves, digestive and

* Many valuable illustrations of the natural laws may be studied in the "Principles of Physiology, applied to the Preservation of Health, and to the Improvement of Physical and Mental Education," 8vo. Edinburgh and London, 1835—*third* edition. We would advise all medical students and practitioners to make this instructive volume the subject of careful and frequent perusal; it abounds with useful observations, physiological and philosophical, the result of its author's extensive experience and intense reflection.

respiratory organs, furnish pleasing sensations directly or indirectly, when exercised in conformity with their nature; and the external senses and internal faculties, when excited, supply the whole remaining perceptions and emotions, which, when combined, constitute life and rational existence. If these were habitually buried in sleep, or constitutionally inactive, life, to all the purposes of enjoyment, might as well be extinct; existence would be reduced to mere vegetation, without consciousness."—" In the *second* place (p. 110), to reap enjoyment in the *greatest quantity*, and to maintain it *most permanently*, the faculties must be gratified *harmoniously*: in other words, if, among the various powers, the *supremacy* belongs to the moral sentiments, then the aim of our habitual conduct must be the attainment of objects suited to gratify them. For example, in pursuing wealth or fame, as the leading object of existence, full gratification is not afforded to the sentiments of benevolence, veneration, and conscientiousness, and, consequently, complete satisfaction cannot be enjoyed; whereas, by seeking knowledge, and dedicating life to the welfare of mankind and obedience to God, in our several vocations, these sentiments will be gratified, and wealth, fame, health, and other advantages will flow in their train, so that the whole mind will rejoice, and its delight will remain permanent. *Thirdly*, to place human happiness on a secure basis, the laws of external creation themselves must accord with the dictates of the moral sentiments, and intellect must be fitted to discover the nature and relations of both, and to direct the conduct in harmony with them."

Mr. C. in the next place, proceeds to make an application of the natural laws to the practical arrangements of life; and, at p. 113, he observes:—If a system of living and occupation were to be framed for human beings, founded on the exposition of their nature now given, it would be something like this—1st, So many hours a day should be dedicated, by every individual in health, to the exercise of his nervous and muscular systems in labour, calculated to give scope to their functions: the reward of obeying this requisite of man's nature would be health, and joyous animal existence—the punishment of its neglect is disease, low spirits, and death. 2d, So many hours a day should be spent in the sedulous employment of the knowing and reflecting faculties—in studying the qualities of external objects and their relations, also the nature of animated beings and their relations, not with the view of accumulating mere abstract and barren knowledge, but of enjoying the positive pleasure of mental activity, and of turning every discovery to account, as a means of increasing happiness or alleviating misery. 3d, So many hours a day should be devoted to the cultivation and gratification of our moral and religious sentiments, that is to say, in exercising these in harmony with intellect, and especially in acquiring the habit of admiring, loving, and yielding obedience to the Creator and his institutions; intellect is barren of practical fruit, however rich it may be in knowledge, until it is fired, and prompted to act, by moral sentiment; it ought, besides, to be regularly exercised in arts, science, philosophy, and observation. 4th, A certain portion of time should be employed in taking food, in sleep, and in innocent amusement. At p. 121, we find the following observations.

"The grand sources of human suffering, at present, arise from bodily disease and mental anxiety, and these may be traced to infringement, through ignorance or otherwise, of physical, organic, moral, or intellectual laws, which, when expounded, appear in themselves calculated to promote the happiness of the race. It may be supposed that, according to this view, as knowledge accumulates, enjoyment will decrease; but ample provision has been made against this event, by withholding intuition from each generation as it appears on the stage. Each successive age must acquire knowledge for itself; and, provided ideas are new to us, and suited to the faculties, the pleasure of acquiring them from instructors, is only second to that of discovering them for ourselves; and, probably, countless ages may elapse before *all* the facts and relations of Nature shall have been explored, and the possibility of discovery exhausted; if the universe be infinite, knowledge can never be complete."

We have now arrived at a stage of Mr. Combe's investigation to which we would particularly direct the attention of physicians and physiological inquirers. Here he proposes to consider some of the evils which have afflicted the human race, and to ascertain whether these have proceeded from abuses of institutions, benevolent and wise in themselves, and calculated, when observed, to promote the happiness of man, or from a constitution of nature so defective that we cannot supply its imperfections, or so vicious that we can neither rectify nor improve its qualities. He subdivides this branch of his inquiry into three sections —on the calamities arising from infringement of the physical laws—on the evils that befall mankind from infringement of the organic laws—and on the calamities occasioned by infringement of the moral laws—we confine our observations and extracts to the second of these sections, on account of its unfolding principles adapted to promote the extension of medical knowledge and usefulness.

An organized being is one which derives its existence from a previously existing organized being, that subsists on food, grows, attains maturity, decays, and dies. Whatever the ultimate object of the Creator, in constituting organized beings, may be, it will scarcely be denied, says Mr. C., that part of his design is, that they should enjoy their existence; and, if so, the object of every particular part of their structure ought to be found to conduce to this end. The *first* law, then, that must be obeyed, to render an organized being perfect in its kind, is, that the germ from which it springs shall be complete in all its parts, and sound in its whole constitution: the *second* is, that the moment it is ushered into life, and as long as it continues to live, it shall be supplied with food, light, air, and every physical aliment necessary for its support: and the *third* law is, that it shall duly exercise its functions. Where all these laws are obeyed, the being should enjoy pleasure from its organized frame, if its Creator is benevolent; and its constitution should be so adapted to its circumstances, as to admit of obedience to these laws, if its Creator is wise and powerful. Is there, then, no such phenomenon on earth, as a human being existing in full possession of organic vigour, from birth till advanced age, when the organized system is fairly worn out? Numberless examples of this kind have occurred, and they show to demonstration, that the corporeal frame of man is so constituted as to admit the *possibility* of his enjoying organic health and vigour during the whole period of a long life; and Mr. C. concludes, since a natural law never admits of an exception, this excellent health would not occur in any individuals unless it were fairly within the capabilities of the race. Holding this doctrine as established, he proceeds to inquire into the causes why these advantages are not universal. He begins, at p. 138, with repeating—

"That the germ of the infant being must be complete in all its parts, and perfectly sound in its condition, as an indispensable requisite to its vigorous development and full enjoyment of existence. If the corn that is sown be weak, wasted, and damaged, the plants that spring from it will be feeble, and liable to speedy decay. The same law holds in the animal kingdom; and, I would ask, has it hitherto been observed by man? It is notorious that it has not: indeed, its existence has been altogether unknown, or in a very high degree disregarded by human beings. The feeble, the sickly, the exhausted with age, and the incompletely developed, through extreme youth, marry, and without the least compunction regarding the organization which they shall transmit to their offspring, send into the world miserable beings, the very rudiments of whose existence are tainted with disease. If we trace such conduct to its source, we shall find it to originate either in animal propensity, or in intellectual ignorance, or more frequently in both. The inspiring motives are generally mere sensual appetite, avarice or ambition, operating in the absence of all just conceptions of the impending evils. The punishment of this offence is debility and pain, transmitted to the children, and reflected back in anxiety and sorrow on the parents. Still the great point to be kept in view is, that these miseries are not legitimate consequences of *observance* of the organic laws, but the direct chastisement of their infringement. These laws are unbending, and admit of no exception; they must be fulfilled, or the penalties of dissolution will follow. From observation, I am convinced that the union of certain temperaments and combinations of mental organs, in the parents, are highly conducive to health, talent, and morality, in the offspring, and *vice versâ;* and, that these conditions may be discovered and taught with far greater certainty, facility, and advantage, than is generally imagined. It will be time enough to conclude that men are naturally incapable of obedience to the organic laws, when, after their intellectual faculties and moral sentiments have been trained to observance of the Creator's institutions, as at once their duty, their interest, and a grand source of their enjoyment, they shall be found to continue to rebel. The *second* requires nutriment to be supplied in due quantity and of a suitable kind, with free air, light, cleanliness, and attention to every physical arrangement by which the functions of the body may be favoured or impaired. Have mankind obeyed or neglected this institution? I need scarcely answer the question. To be able to obey institutions, we must first know them: before we can know the organic constitution of our body, we must study that constitution, and the study of the human constitution is anatomy and physiology: before we can become acquainted with its relations to external objects, we must learn the existence and qualities of these objects as unfolded by chemistry, natural history, and natural philosophy, and compare them with the constitution of the body. When we have fulfilled these conditions, we shall be better able to discover the laws which the Creator has instituted in regard to our organic system. That all our functions shall be duly exercised, is the *third* organic law: has this law been properly observed by mankind? Many persons are able, from experience, to attest the severity of the punishment that follows from neglecting to exercise the nervous and muscular systems, in the lassitude, indigestion, irritability, debility, and general uneasiness that attend a sedentary and inactive life."

The brain is the fountain of nervous energy to the whole body; and, it is Mr. C.'s opinion, that many persons are habitual invalids, without actually labouring under any ordinary recognized disease, solely from defective or irregular exercise of the nervous system. In such cases, not only the mind, in its feelings and intellectual capacities, suffers debility, but all the functions of the body participate in its languor, because all of them receive a diminished and vitiated supply of the nervous stimulus, a due share of which is essential to their healthy action. The mode of increasing the strength and energy of any organ and function, is to exercise them regularly and judiciously according to the laws of their constitution. The brain is the organ of the mind; different parts of it manifest distinct faculties; and the power of manifestation in regard to each is proportionate, *cæteris paribus*, to the size and activity of the organ. The brain partakes of the general qualities of the organised system, and is strengthened by the same means as the other organs. When the muscles are called into vivacious activity, an increased influx of blood and nervous stimulus takes place in them, and their vessels and fibres become at once larger, firmer, and more susceptible of action. Thought and feeling are to the brain what bodily exercise is to the muscles; they put it in motion, and cause increased action in its blood-vessels, and an augmented elaboration of nervous energy. Mr. C. illustrates this position by the case of a female, in whom a part of the skull had been removed. Her brain was motionless and lying within the cranium, when she was in a dreamless sleep: it was in motion and protruding without the skull when she was agitated by dreams. It protruded more in dreams reported by herself to be vivid, and still more so when she was perfectly awake, and especially if engaged in action, thought, or sprightly conversation. Nearly twenty years ago, we had frequent opportunities of witnessing similar phenomena in a robust young man, who lost a considerable portion of his skull by an accident which had almost proved mortal. When excited by pain, fear, or anger, his brain protruded greatly, so as sometimes to disturb the dressings, which were necessarily applied loosely, and it throbbed tumultuously in accordance with the arterial pulsations.

Those parts of the brain which manifest the feelings constitute by far the largest portions of it, and they are best exercised by discharging the active duties of life and religion: the parts which manifest the intellect are smaller, and are exercised by the application of the understanding in practical business in the arts, sciences, or literature. The *first* step, therefore, towards establishing the regular exercise of the brain, is to educate and train the mental faculties in youth; and the *second* is, to place the individual habitually in circumstances demanding the fulfilment of useful and important duties. Different modifications of the nervous energy elaborated by the brain, appear to take place according to the mode in which the faculties and their organs are affected. Thus, when misfortune and disgrace impend over us, some of the cerebral organs are painfully excited, and transmit an impaired, or positively noxious, nervous influence to the heart, stomach, intestines, and thence to the rest of the body: digestion is then deranged, the pulse becomes feeble and irregular, and the whole corporeal system wastes. On the other hand, when the cerebral organs are agreeably affected, a benign and vivifying nervous influence pervades the frame, and all the functions of the body are performed with increased pleasure and success. It is a law, that the quantum of nervous energy increases with the number of cerebral organs roused to activity; and history, as well as experience, abounds with confirmations of this doctrine. The more elevated the objects of our study, the higher are the mental organs which are exercised; and the higher the organs, the more pure and intense is the pleasure: hence, a vivacious and regularly-supported excitement of the moral sentiments and intellect, is, by the organic law, highly conducive to health and organic vigour. In the fact of a living animal being able to retain life in an oven that will bake dead flesh, we see an illustration of the organic law arising above the purely physical; and, in the circumstance of the moral and intellectual organs transmitting the most favourable nervous influence to the whole bodily system, we have an example of the moral and intellectual law rising higher than the merely organic. Mr. Combe elucidates the foregoing principles, which are of great practical importance, by a few actual cases. We give some of these in the following extract:—

"Two or three centuries ago, various cities in Europe were depopulated by the plague; and, in particular, London was visited by an awful mortality from this cause. The people of that age attributed this scourge to the inscrutable decrees of Providence, and some to the magnitude of the nation's moral iniquities: but, according to the views now presented, it must have arisen from infringement of the *organic laws,* and been intended to enforce stricter obedience to them in future. Its causes and objects, when clearly analyzed, appear to have had no *direct* reference to the moral condition of the people. The facts recorded in history exactly correspond with the theory here propounded. In the fifteenth century, the floors of the houses being commonly of clay, and strewed with rushes or straw, it is loathsome to think of the filth collected in the hovels of the common people, and sometimes in the lodgings even of the superior ranks, from spoiled milk, beer, grease, fragments of bread, flesh, bones, spittle, excrements of cats, dogs, and other animals. From similar causes arose the plague and sweating-sickness of London, which, in this respect, resembled Paris and other towns of any magnitude, in those times. The streets of the former metropolis were excessively narrow, the habits of the people dirty, and no adequate provision was made for removing the filth unavoidably produced by a dense population. The great fire in that city, which happened soon after the pestilence, afforded an opportunity of remedying, in some degree, the narrowness of the streets; habits of increasing cleanliness abated the filth; and these changes brought the people into a closer obedience to the organic laws, and no plague has since returned."—Obviously, therefore, if Mr. Combe's theory be the right one, obedience to the natural laws will prove a better security from "plague, pestilence, and famine," than all the expedients of quarantine and political economy which have hitherto been invented.

Having traced bodily suffering in individuals to neglect of, or opposition to, the organic laws, Mr. C. next adverts to another set of calamities, which may be called social miseries, and obviously spring from the same causes. This leads him to expatiate on the transmission of hereditary qualities, on neglect of the organic laws in marriage, on the choice of servants, and on death, which he represents as a natural institution, alike wise and benevolent. For an exhibition of the facts and inductions on which this doctrine is founded, we must refer our readers to the 217—235 pages of his volume, where they will find that Mr. C.'s views on this, as on every other subject, are explained with singular perspicuity and comprehensiveness, with an earnestness and candour unusually persuasive.

With much reluctance, we pass Mr. C.'s truly interesting observations on the calamities arising from infringement of the moral law; on punishment as inflicted under the natural laws, and its moral advantages; on the combined operation of the natural laws, and their influence on the happiness of individuals; and on the relation between science and the Sacred Writings. Our limits reduce us to this necessity: we will, therefore, bring this article to a conclusion with some extracts and reasonings of his on the "transmission of hereditary tendencies," giving preference to this theme because of its relation to physiological knowledge.

Physiologists are agreed, that a vigorous and healthy state of body in the parents communicates existence in the most perfect state to their offspring, and *vice versâ*; and many observers of mankind, as well as medical authors, have remarked the transmission, by hereditary descent, of mental talents and dispositions. These positions are adopted by Mr. Combe, and he confirms them with the authority of reputable writers as well as with his own extensive and diversified experience. Mental talents and dispositions are determined by the size and constitution of the brain, which is a portion of man's organized system; and, as such, it is subject to the organic laws, by one of which its qualities are transmitted by hereditary descent. This law, however faint and obscure it may appear in individual cases, becomes absolutely undeniable in nations. When we place a collection of Hindoo, Carib, Negro, New Holland, North America, and European skulls in juxta-position, we perceive a national form and combination of organs in each, actually obtruding itself upon our notice, and corresponding with the mental characters of the respective tribes: the cerebral development of one tribe is seen to differ as widely from that of another, as the European mind does from that of the New Hollander. Here, then, each Hindoo, Chinese, Negro, and Carib, obviously inherits from his parents a certain general type of head; and so does each European. If, therefore, the general forms and proportions are thus so palpably transmitted, can we doubt that the individual varieties follow the same rule, modified slightly by causes peculiar to the parents of the individual? The differences of national *character* are equally conspicuous as those of national *brains*, and it is surprising how permanently both endure. Now, if form, size, and constitution of brain, are thus transmitted from parents to children; if these cerebral conditions determine natural mental talents and dispositions, which in their turn exercise the greatest influence over the happiness of individuals through the whole of life; it becomes extremely important to discover the laws according to which this transmission takes place. At the first aspect of the question, three principles present themselves to our consideration. Either, in the *first* place, the constitution and qualities of brain, which the parents themselves inherited at birth, are transmitted absolutely, so that the children, sex following sex, are exact copies, without variation or modification, of the one parent or the other; or, *secondly,* the natural and inherent qualities of the father and mother combine, and are transmitted in a modified form to the offspring; or, *thirdly,* the qualities of the children are determined jointly by the constitution of the stock, and by the faculties which predominate in power and activity in the parents, at the particular time when the organic existence of each child commences. Experience shews that the *first* cannot be the law; for, a real law of nature admits of no exceptions, and it is a well-established fact, that the minds of children are not exact copies, without variation or modification, of those of the parents, sex following sex. Neither can the *second* be the law, because it is equally certain that the minds of children, although *sometimes,* are *not always,* in talents and dispositions exact blended reproductions of those of the father and mother. If this law prevailed, no child would be a copy of the father, none a copy of the mother, nor of any collateral relation, but each would be invariably a compound of the two parents, and all the children would be exactly alike, sex alone excepted. Experience shews that this is not the law: what, then, does experience say to the *third* idea, that the mental character of each child is determined by the particular qualities of the stock, combined with those which predominated in the parents when its existence commenced.

Having previously adverted to the influence of the stock, Mr. C. here (p. 182) illustrates that of the condition of the parents, when existence is communicated. One striking and undeniable proof of the effect on the character and dispositions of children, produced by the form of brain transmitted to them by hereditary descent, is to be found in the progeny of marriages betwixt Europeans, whose brains possess a favourable development of the moral and intellectual organs, and Hindoos and native Americans, whose brains are inferior. All authors agree, and report the circumstance as singularly striking, that the children of such unions are decidedly superior in mental qualities to the native, while they are still inferior to their European parent. So much is this the case in Hindostan, that several writers have already pointed to the mixed race there, as obviously destined to become the future sovereigns of India. These individuals inherit from the native parent a certain adaptation to the climate, and, from the European parent, a higher development of brain—the two combined constituting their superiority. Another illustration of the same law is found in the fact, that when two persons marry very young, the eldest of their children inherits a less favourable development of the moral and intellectual organs, than those produced in more mature age. Generally, in men, the animal organs are most vigorous in early life, and this energy appears to cause them to be then most readily transmitted to offspring. Indeed, says Mr. C. it is difficult to account for the wide varieties in the form of the brain, in children of the same family, unless on the principle, that the organs which predominate in vigour and activity in the parents, at the time when existence is communicated, determine the tendency of corresponding organs to develop themselves largely in the children. Now, if this be really the law of nature, then parents, in whom the animal propensities are habitually active, will transmit the organs of these propensities in a state of high development and excitement to their children: and, in those in whom the moral and intellectual organs exist in supreme vigour, will transmit these in greatest perfection. This view is in harmony with the fact, that children generally, though not universally, resemble their parents in their mental qualities; because, the largest organs being naturally the most active, the general and habitual state of the parents will be determined by those which predominate in size in their own brains; and, on the principle that predominance in activity and energy causes the transmission of similar qualities to the offspring, the children will, in this way, very generally resemble the parents. But they will not always do so; because even very inferior characters, in whom the moral and intellectual organs are deficient, may be occasionally exposed to external influences, which, for the time, may excite these organs to unwonted vivacity, and, according to the rule now explained, a child, dating its existence from that period, may inherit a higher constitution of mind, and a co-existent higher organization of brain, than the parents. Or a person with an excellent moral development may, by some particular occurrence, have his animal propensities roused to unwonted vigour, and his moral sentiments thrown, for the time, into the shade; and any offspring connected with this condition would prove inferior to himself in the development of the moral organs, and greatly surpass him in the size of those of the propensities. Mr. C. does not present these views as ascertained science, but as inferences, strongly supported by facts, and consistent with known phenomena: if we believe them to be true, they will powerfully strengthen the motives for preserving the *habitual supremacy* of the moral sentiments and intellect, when, by so doing, improved moral and intellectual capacities may be conferred on offspring. In fine, since activity in the faculties is the fountain of enjoyment, the whole constitution of nature is designedly framed to support them in ceaseless action. What scope, then, for observation, reflection, the exercise of moral sentiments, and the regulation of animal impulse, does not this picture of nature present!

Mr. Combe believes that his essay contains views of the constitution, condition, and prospects of man, which deserve attention; but, with characteristic modesty, he lays no claim to originality of conception, stating that the only novelty in his pages, respects the relations which substantiated truths hold to each other. Physical laws of nature, he remarks, affecting our physical condition, as well as regulating the whole material system of the universe, are universally acknowledged, and form the elements of natural philosophy and chemical science. Physiologists, and medical practitioners, admit the existence of *organic* laws; and the sciences of government, legislation, education, indeed our whole train of conduct through life, proceed upon the admission of laws in morals. Accordingly, the laws of Nature have formed an interesting subject of inquiry to philosophers of all ages; but no author has hitherto attempted to point out, in a combined and systematic shape, the relations between these laws and the constitution of man—nor has any preceding writer unfolded the independent operation of the several natural laws, and the practical consequences which follow from this fact: nevertheless, all this must be done before our knowledge of them can be beneficially applied. The great object of Mr. C.'s book is to exhibit these relations and results, with a view to the improvement of education, and the regulation of individual and national conduct; and, with some right to entertain an opinion on such questions, we feel justified in stating, with perfect sincerity, that his essay has been crowned with extraordinary success.

Having considered man as a *physical* being, and adverted to the adaptation of his constitution to the physical laws of creation—having viewed him as an *organized* being, and traced the relations of his organic structure to his external circumstances—having taken a survey of his *faculties* as an animal, moral, and intellectual being, with their uses, and the forms of their abuses—having contrasted these faculties with each other, and discovered the supremacy of the moral sentiments and intellect; and having compared his faculties with *external objects*, in order to ascertain what provision has been made by the Creator for their gratification—having accomplished these arduous and important ends, it is manifest that Mr. Combe must have investigated, and, as we think, clearly established, many elementary doctrines, possessing an absolute and constitutive bearing upon the foundations of those sciences, whereof medicine is the aggregate. Heretofore, this has been essentially crippled in its advances, by the oversight of neglecting to make the acquisition of mathematical, metaphysical, and ethical knowledge, an integral and indispensable requisite in completing the evidences of qualification, to recognize the actual characters of disease, and to discriminate the therapeutical agents by which they can be removed. Whoever, therefore, encourages a desire to win distinction as an intelligent physician, should zealously cultivate his knowing and judging faculties; and, with a view to promote his meritorious exertions, we would solicit him to study, particularly and perfectly, Mr. C.'s excellent "*Constitution of Man.*"

